# Supplementary material for: Structural basis for matriglycan synthesis by the LARGE1 dual glycosyltransferase
Source: PLoS One. 2022 Dec 13;17(12):e0278713. doi: 10.1371/journal.pone.0278713 (PMC9746966; doi:10.1371/journal.pone.0278713)

Raw image for figure 7b

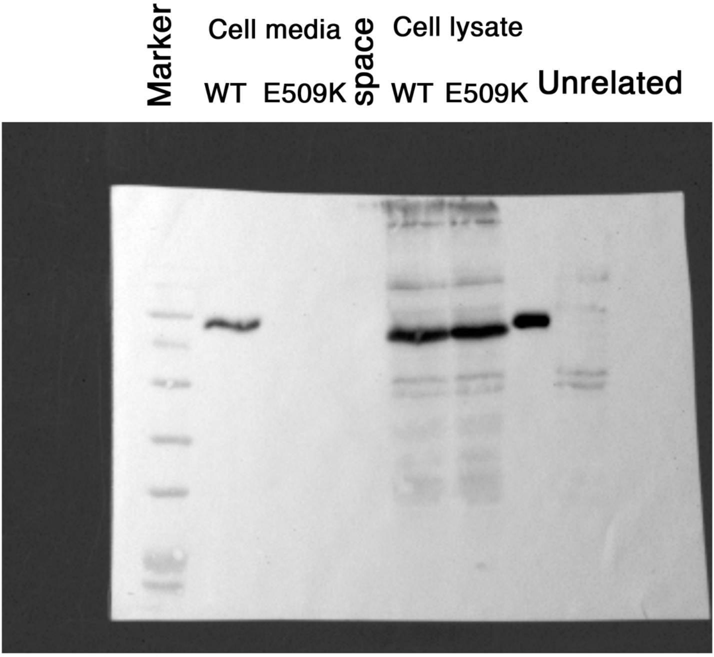

Raw image for figure 7c

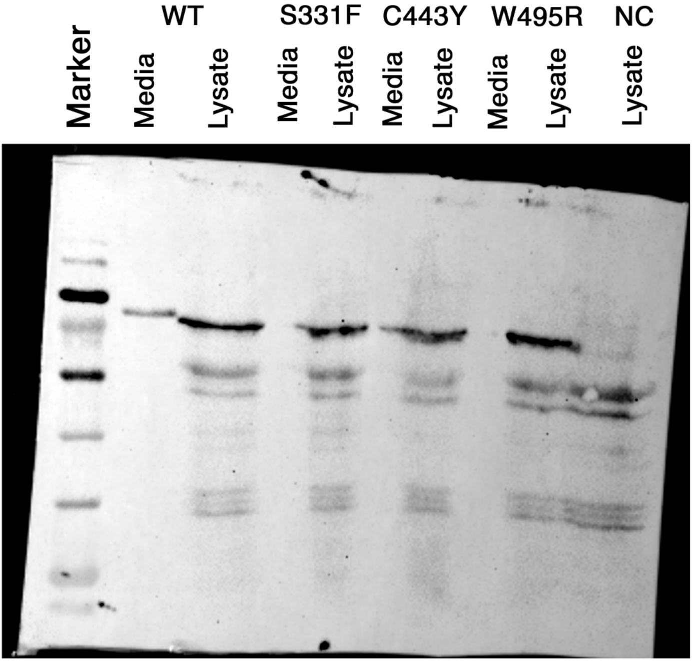

Supplement: S1 Raw images — (PDF) [file pone.0278713.s001.pdf]
